# Analysis of amyloid beta oligomers by cyclic ion mobility-mass spectrometry

**DOI:** 10.1007/s00216-026-06349-w

**Published:** 2026-01-31

**Authors:** Mikuláš Vlk, Alexander Muck, John A. Hey, Jean F. Schaefer, Martin Hubálek, Josef Cvačka

**Affiliations:** 1 Department of Analytical Chemistry, Faculty of Science, Charles University, 128 00, 2030/8 Prague, Czech Republic; 2 Institute of Organic Chemistry and Biochemistry, Czech Academy of Sciences, Flemingovo náměstí 542/2, 16600 Prague, Czech Republic; 3 Waters Corporation, Stamford Avenue, Altrincham Road, Wilmslow, SK9 4AX UK; 4 Alzheon, Inc., 111 Speen Street, Framingham, MA 01701 USA

**Keywords:** Aβ(1–42) oligomers, Cone voltage, In-source activation, Ion transmission, Collision-induced unfolding

## Abstract

Amyloid-beta (Aβ) oligomers are key contributors to the pathology and progression of Alzheimer’s disease (AD), making their characterization essential for understanding aggregation processes and developing potential therapeutic strategies. This study provides a systematic framework for analyzing Aβ(1–42) oligomers in vitro using cyclic ion mobility-mass spectrometry (cIMS). Compared to previous generations of traveling wave ion mobility (TWIM) devices, the cIMS platform offers superior resolution through its scalable ion mobility path length. However, the multistage character of the cIMS platform requires thorough investigation of parameters affecting oligomer transmission and activation to ensure reliable analysis of labile and dynamic systems such as Aβ oligomers. Our findings highlight the critical influence of cone voltage (CV) on in-source ion activation, subsequent structural changes, and oligomer detection. By balancing CV, we achieved detection of a broad range of oligomeric species while limiting their activation and maximizing signal intensity. Moreover, we present the first comprehensive set of optimized ion optics and ion mobility parameters that enable effective transmission and separation of oligomer Aβ(1–42) ions. Using the optimized method, we successfully detected a spectrum of Aβ(1–42) oligomers ranging from dimers to dodecamers. Additionally, the method was applied in collision-induced unfolding experiments, revealing size-dependent conformational transitions proving its applicability. This optimized cIMS methodology establishes a foundation for future studies on Aβ(1–42) aggregation mechanisms, AD pathogenesis, and therapeutic applications. Furthermore, our results offer valuable insights into cIMS instrument tuning, with potential applications in the analysis of other complex biological systems.

**Graphical Abstract:**

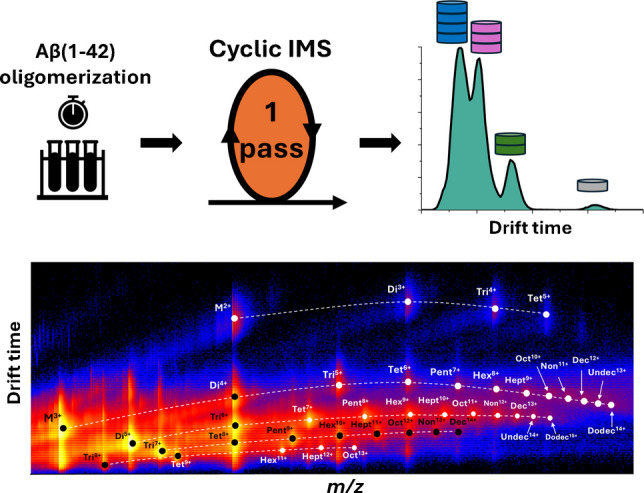

**Supplementary Information:**

The online version contains supplementary material available at 10.1007/s00216-026-06349-w.

## Introduction

Alzheimer’s disease is the most widespread neurodegenerative disease worldwide [1, 2]. The amyloid hypothesis describes the critical role of amyloid beta aggregation followed by accumulation, which triggers a neurotoxic cascade, leading to impaired synaptic communication, inflammation, neuronal death, and brain atrophy [3–5]. Amyloid-beta (Aβ) oligomers have been shown to be neurotoxic [6–10] and are one of the hallmarks of AD [11, 12]. Recent studies also highlight that Aβ oligomers can interact with tau protein, enhancing its hyperphosphorylation [13] and accelerating cognitive decline in AD patients [14]. Moreover, the interactions between Aβ oligomers and tau contribute to changes in gene expression associated with AD [15]. These gene expression changes include alterations in pathways related to synaptic plasticity, inflammation, and cell survival [14].

Thorough characterization of Aβ oligomers is essential for understanding the oligomerization process and potentially evaluating disease progression. This is particularly important as Lecanemab, the first FDA-approved treatment, targets large oligomers as the basis for its efficacy in AD [16] and the majority of phase 3 clinical trials are anti-amyloid [17]. Moreover, the aggregation of Aβ(1–42) in vitro is a complex sequential interplay between oligomer and fibril formation, which depends on environmental factors like incubation time, concentration, and temperature [18–21]. This two-step process, involving oligomer formation followed by conversion into fibrillar aggregates, occurs on a minute to hour timescale, depending on conditions [22, 23].

Ion mobility-mass spectrometry (IM-MS) was successfully used to study in vitro Aβ(1–42) oligomerization [24, 25] and inhibitor binding [26–28]. Some studies report detection up to dodecamer [25, 29] while others show a prevalent formation of dimers, trimers, and hexamers [30, 31].

The parameters of the IM-MS method significantly influence the effectiveness of non-covalent complex transmission and their structure. One of the key parameters is the entrance potential, moving ions formed by (nano)electrospray ionization across the pressure gradient between the nearly atmospheric source region and the ion guide with lower pressure [32, 33]. This parameter is referred to as cone voltage (CV) in the context of Waters mass spectrometers [34–36]. At increased CVs, the ions are accelerated and heated by collisions with residual gas molecules within the Step-Wave travelling wave ion guide. The ion heating subsequently leads to desolvation, declustering, and, at excessive CVs, fragmentation [34, 37, 38]. In contrast, elevated pressure in the source region promotes collisional cooling by transferring internal energy from ions to neutral gas molecules, as well as through adiabatic expansion [39, 40]. This reduces the ions’ radial and axial speed and limits desolvation, potentially causing peak broadening [33]. In earlier generations of mass spectrometers, source pressure modulation was performed using a restricting valve on the roughing pump line [32, 41]. However, previous research proved that the cyclic ion mobility mass spectrometer [42] is capable of transmission of large non-covalent assemblies with masses in the megadalton range [43] without the need for source pressure adjustment, based on changes to the source hardware design (e.g., larger cone diameter allowing for higher pressure values around 2–5 mBar). The cIMS device also offers a longer travelling wave ion mobility (TWIM) flight path (97.6 cm) compared to the previous generation of TWIM devices, such as Synapt G2(Si) (25.5 cm), offering higher resolution. Moreover, it provides superb versatility, including ion selection based on both mobility and mass as well as IMS^n^ experiments and scalable (single-pass or multi-pass) ion mobility separations. While the present work focuses mainly on a single-pass approach for method optimization, these parameters are transferable to multi-pass workflows. Due to the added complexity of the cIMS instrument, a detailed optimization of the instrument parameters is required for efficient detection of labile Aβ(1–42) oligomers. It was shown that cIMS ion optics could transmit labile species such as adenine cation radicals [44] and cytochrome c 7 + conformer [45]. Previous research has not yet provided a systematic experimental framework for Aβ(1–42) oligomer analysis by cIMS, which could facilitate progress in studying inhibitor binding, oligomerization processes, or disease progression.

Our study aims to provide a systematic toolbox for researchers by identifying and describing those critical instrumental parameters that influence the successful detection of Aβ(1–42) in vitro by cIMS. The optimized method can serve as a foundation for future research, including complex multi-pass experiments. We highlight the importance of in-source parameters such as cone voltage, which directly affects ion activation, structural integrity, and detection of the oligomers. Furthermore, we describe parameters in each part of the instrument’s ion optics that influence the sensitivity of oligomer detection.

## Methods

### Chemicals

The Aβ(1–42) peptide (> 97%) was purchased from rPeptide (Watkinsville, USA). Water (LC-MS grade) was purchased from Fisher Chemical (Waltham, USA). Ammonia solution LC-MS grade (25%) was obtained from Merck (Darmstadt, Germany). Caesium iodide (99.999%) and 1,1,1,3,3,3-hexafluoro-2-propanol (HFIP) (> 99%) were purchased from Sigma Aldrich (St. Louis, USA).

### Sample preparation

The Aβ(1–42) peptide was dissolved in HFIP + 3% NH_3_ (*v/v*) to form a 1.0 mg/ml solution and sonicated in the glass vial for 5 min according to the procedure published by Stark et al. [46]. Aliquots of 25 µl were dispensed into 0.5-ml Protein LoBind Tubes (Eppendorf AG, Hamburg, Germany), and the solvent was removed using a Labconco centrifugal vacuum evaporator (Kansas City, MI, USA). The aliquots were stored at −20 °C. For further experiments, the peptide film was dissolved in water to 22 µmol/l, vortexed, and incubated for 24 h at 21 °C to induce oligomer formation. The incubated samples were then stored on ice prior to analysis. All sample preparation steps, including incubation, were performed without controlling illumination conditions, as our results showed that incubating samples in complete darkness had no measurable effect on oligomer formation.

### Ion mobility-mass spectrometry

Mass spectrometry measurements were performed on a SELECT SERIES Cyclic IMS mass spectrometer (Waters Corp., Milford, USA) fitted with surface-induced dissociation and electron-capture dissociation (ECD) (MSIon, Portland, Oregon, USA) cells [42]. Mass calibration was performed using 1.0 mg/ml CsI solution in the *m/z* 100–8000 range. Samples were introduced by an in-house-made static nanoelectrospray ion source. GELoader tips (Eppendorf, Hamburg, Germany) were used for the injection of 4 µl of the sample into a borosilicate nanoelectrospray emitter with an orifice diameter of ~1 μm which was pulled in-house on a P1000 capillary puller (Sutter Instrument, Novato, USA) using borosilicate capillary glass with 1 mm and 0.58 mm external and internal diameters. The capillary with the sample was inserted into an in-house 3D printed capillary holder and centrifuged at 1000 g for 5 s to transfer the sample solution into the tip of the emitter. A stainless-steel filament was inserted into the capillary to introduce the spray voltage of 0.7–1.4 kV with the ion source temperature set to 20 °C. The ECD cell was set into a flythrough mode with the following settings: L1 −0.5, L2 −4.0, LM3 0.0, L4 0.4, FB 0.3, LM5 −7.0, L6 −5.0, L7 0.4 Volts, respectively. The complete list of instrumental parameters can be found in Supplementary Tables S1 and S2.

### Data processing

Data analysis was performed in MassLynx v4.2 and DriftScope v3.0 software (Waters Corp., Milford, USA). Smoothing was applied to the recorded spectra with the following settings: smooth window, 3; number of smooths, 2; smoothing method, mean. Collision-induced unfolding data was processed and visualized by CIUSuite 2 software package [47]. Gaussian fitting was performed using the Peak Analyzer tool in OriginPro with the following settings: peak-finding method, 2nd derivative; peak-filtering threshold, 1% of peak height; curve fitting function, biGaussian; iteration tolerance, 1 × 10⁻⁹; and maximum number of iterations, 200.

The following abbreviations were used for the annotation of detected oligomers: M, monomer; Di, dimer; Tri, trimer; Tet, tetramer; Pent, pentamer; Hex, hexamer; Oct, octamer; Non, nonamer; Dec, decamer; Undec, undecamer; Dodec, dodecamer.

## Results and discussion

### Ion transmission optimization

The first step of the method development was to ensure controlled formation of Aβ(1–42) oligomers in vitro in water. In order to break down pre-existing aggregates, we employed HFIP and NH_3_ treatment, a procedure commonly used to disaggregate Aβ peptides [20, 25]. The effect of sample incubation on oligomer formation is shown in Supplementary Figure S1.

While sample preparation helps control oligomer formation in solution, our main focus was to create an optimized cIMS method. Both ion source settings and ion optics of the mass spectrometer had to be tuned to enable transmission of the non-covalent complex ions and to maintain their structural integrity. In the following section, we aim to describe key parameters affecting ion transmission and their impact on oligomer detection.

Native mass spectrometry analysis requires thorough optimization of instrumental parameters to preserve non-covalent interactions and folded conformation of studied biomolecules [43]. Although nanoelectrospray ionization enables sufficiently soft ionization of non-covalent complex ions, the optimal transmission of Aβ(1–42) oligomer ions requires properly set voltages guiding ions along the ion path. Fine-tuning of ion optics settings is therefore necessary to prevent the disassembly of oligomeric forms or their ineffective transmission, which could alter the quantitative data. In this section, we present the optimization results of key parameters to maximize ion transmission while preserving the native state of the Aβ(1–42) oligomers.

**Fig. 1:**
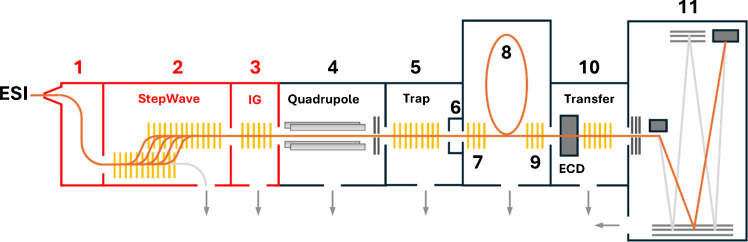
Schematic representation of the cyclic ion mobility mass spectrometer. (1) Ion block with sample cone, (2) StepWave ion guide, (3) segmented quadrupole ion guide, (4) quadrupole, (5) trap collision cell, (6) helium cell, (7) pre-array store, (8) cIMS cell (racetrack), (9) post-array store, (10) segmented quadrupole transfer collision cell with optional electron-capture dissociation, (11) TOF detector. The source region is highlighted in red

The schematic illustration of the cyclic ion mobility mass spectrometer is shown in Fig. 1. Analytes are ionized in the nanoelectrospray source and transferred into the instrument through the sample cone. Next, a dual off-axis ion guide assembly (StepWave) filters out neutral components and focuses the charged particles to enhance ion transmission, followed by a segmented quadrupole ion guide. From here, ions go through a quadrupole filter operated in RF mode, followed by the first collision cell (trap), which accumulates ions before their injection into the ion mobility cell through an elevated pressure helium cell.

The ion mobility region consists of a pre-array store that can be used to perform IM^2^ re-isolation of IM-separated ions. The vital part of the IM device, the multifunctional array, is situated at the bottom of the cyclic cell and functions as a multidirectional ion switch. It can guide the ions directly toward the transfer region equipped with the ECD cell and detector to bypass the cyclic IMS in a standard TOF operation mode or guide them perpendicularly into the IM separator. Several modes of the multifunctional array enable guiding the ions in a perpendicular direction in the closed-loop electrode array (racetrack) for ion mobility separation, back to the pre-array store, or to the time-of-flight detector. This allows for simple single/multi-pass sequences or more complex IMS^ⁿ^ experiments.

We employed a single-pass method consisting of inject, separate, and eject steps. A detailed description of the timing of the method can be found in Supplementary Table S2.

### Effect of cone voltage

Several potentials of the source region are responsible for the acceleration of ions formed in the nanoelectrospray into the ion optics and facilitate their transfer from the atmospheric pressure region into the low-pressure regions of the mass spectrometer. Modern ESI sources are required to provide high sensitivity, which in most cases is associated with sampling a larger volume of the ambient environment. The highest effect influencing the sensitivity of native assemblies can be found in the intermediate segment where atmospheric pressure is reduced into the source vacuum. Given the importance of this stage in retaining the native structure of analyzed compounds, we focused on a detailed description of the effects of CV on Aβ(1–42) oligomer analysis.

In native mass spectrometry, the cone voltage (CV) is one of the key parameters that must be optimized to balance two critical parameters: effective ion declustering and desolvation and preserving the native conformation and non-covalent interactions. Proper CV adjustment ensures that the Aβ(1–42) oligomers are transferred into the gas phase, retaining their structural integrity, which is essential for getting information on oligomer composition.

One observed effect of increased CV is the shift of the charge state envelope toward ions with lower charge states, resulting in higher mass-to-charge ratios (*m/z*), as shown in Supplementary Figure S2. This shift can be attributed to the following interconnected mechanisms. During the ionization process, ions form clusters with solvent and salts present in the solution. When accelerated by CV, ions with a lower number of charges gain lower kinetic energy, making them less prone to fragmentation and more likely to remain intact as they pass through the ion source optics. Increased CV results in more effective adduct stripping, enhancing the signal intensity of these species.

Simultaneously, ions with higher charge states gain greater kinetic energy when accelerated by the CV. These higher energetic collisions lead to in-source dissociation and signal loss [48, 49]. Furthermore, the increased internal energy gained by the collisions disrupts non-covalent interactions in low *m/z* oligomeric species. This dissociation can generate lower-order oligomer ions with fewer charges, contributing to the observed *m/z* shift. This effect is apparent in Supplementary Figure S2, where the signal of Di^5+^ (*m/z* 1806) present at 20 V CV disappears when the CV is increased to 120 V in favor of M^2+^ (*m/z* 2257) and M^3+^ (*m/z* 1505). Similarly, the Tri^7+^ (*m/z* 1935) species present at 20 V CV is no longer detected at 120 V, while Tri^5+^ (*m/z* 2709) emerges instead.

As a consequence of these effects, the overall charge state distribution shifts toward higher *m/z* values.

**Fig. 2:**
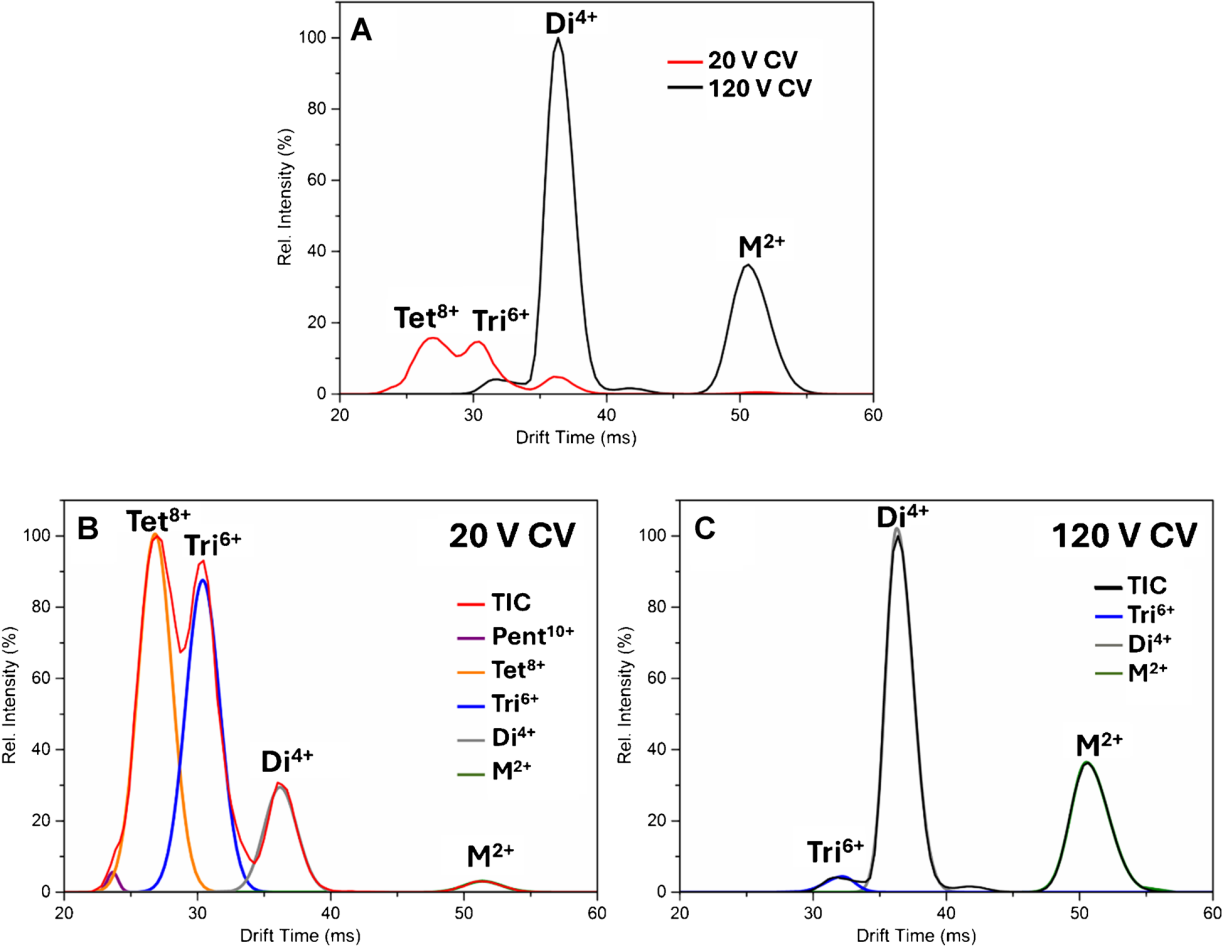
**A** Arrival time distribution of the *m/z* 2257 signal at 20 V CV (red) and 120 V CV (black), showing the distribution shift of detected Aβ(1–42) oligomers. At 20 V CV, the ATD reveals Tet⁸⁺ and Tri⁶⁺ oligomers predominantly, with a minor Di^4^⁺ signal. In contrast, the 120 V CV measurement displays a dominant Di^4^⁺ and M^2^⁺ signal alongside a minor Tri⁶⁺ peak. **B** and **C** Gaussian fitting of ATD at 20 V and 120 V CV, respectively

The mobilograms in Fig. 2 show the effect of CV on the transmission of ions detected at *m/*z 2257. The comparison of arrival time distributions (ATD) at 20 and 120 V in Fig. 2A highlights the differences in signal intensity and oligomer distribution at each voltage. At 20 V, Tet^8+^ and Tri^6+^ were the major detected species, along with minor signals of Di^4+^ and M^2+^. In contrast, at 120 V, sensitivity increased fivefold, with M^2^⁺ and Di^4^⁺ being the dominant species, alongside a minor signal from Tri⁶⁺. At 20 V, the lower collision energy preserves the integrity of larger oligomers such as Tet^8+^ and Tri^6+^. However, the lower energy conditions result in less efficient desolvation and adduct removal, which can limit the sensitivity. At 120 V, higher energy enhances desolvation but causes dissociation of larger oligomers such as Tri^6+^ and Tet^8+^.

To further elucidate the differences in oligomer distribution, Gaussian fitting was applied to each ATD, as shown in Fig. 2B and C. At 20 V, Gaussian fitting revealed one additional peak at 24 ms co-drifting with Tet^8+^. At 120 V, the asymmetric Di^4+^ peak was resolved into two co-drifting components, both corresponding to dimeric species, indicating structural flexibility and the formation of two similar conformations.

**Fig. 3:**
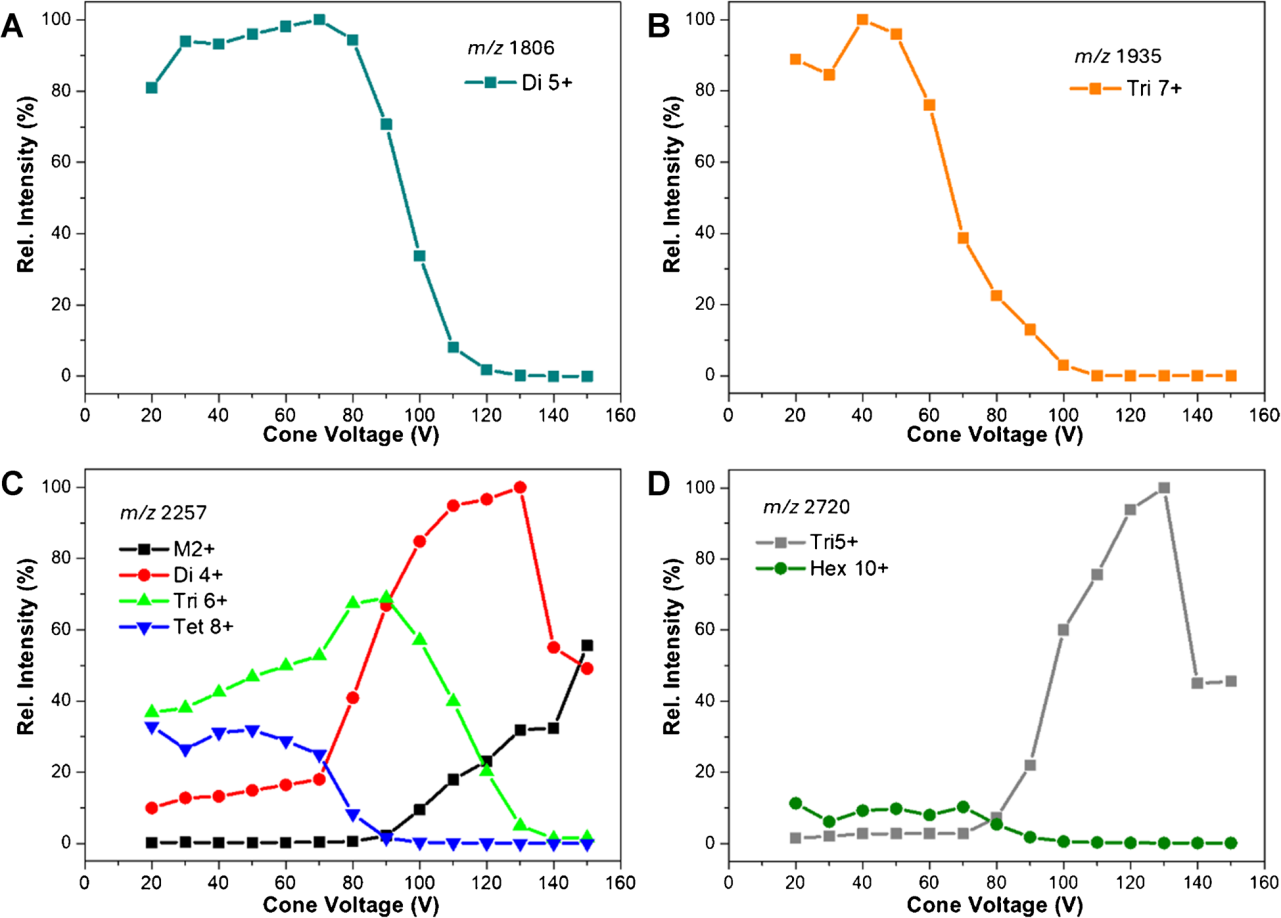
In-source activation of Aβ(1–42) oligomers induced by increasing cone voltage. Relative intensities of oligomer species separated at a given *m*/*z*. The following species were detected: **A** at m/z 1806, the single peak of Di^5+^; **B** At m/z 1935, the single peak of Tri^7+^; **C** at 2257, M^2+^, Di^4+^, Tri^6+^, and Tet^8+^; **D** At m/z 2720, Tet^5+^ and Hex^10+^. The intensity of the detected species is stable up to 60–80 V CV, followed by dissociation into smaller oligomers or monomers. This suggests the dissociation of oligomers caused by in-source activation

The effect of increasing CV on the signal intensities of dimer to hexamer was further investigated. The signals for the Di^5+^ and Tri^7+^ species, as shown in Fig. 3A and B, remain relatively stable at lower CVs, but both show a rapid decrease in intensity at around 50 V and 70 V, respectively. This drop is likely due to the internal energy of the ions reaching a critical level, resulting in dissociation of the non-covalent interaction.

Ion mobility enabled the separation of multiple oligomeric species. Tet^8+^, Di^4+^, and M^2+^ ions were detected at *m/z* 2257, while Hex^10+^ and Tri^5+^ were identified at *m/z* 2720. The Tet^8+^ signal, like D^5+^ and Tri^7+^, begins to drop at 70 V CV. Up to that point, the ratio of Tet^8+^, M^2+^, and Di^4+^ remained stable, followed by an increase in monomer and dimer intensity.

The same pattern was observed in the case of signals at *m/z* 2720, which correspond to Hex^10+^ and Tri^5+^. The intensity of Hex^10+^ starts to decline at around 70 V while the Tri^5+^ increases. This suggests that increasing CV promotes the dissociation of higher-order oligomers into smaller subunits.

Generally, the intensity curve for each oligomer follows a common pattern. Initially, the intensity rises as desolvation and declustering improve, reaching a plateau under optimal conditions for the given oligomer. Subsequently, the signal gradually declines as the internal energy of the oligomer ions reaches a critical value, leading to the dissociation of non-covalent interactions and fragmentation. Comparing the CV at which the signal intensity decreases to 50% of its maximum during the descending phase (CV_50_) provides valuable insights. The corresponding CV_50_ values are listed in Table 1.

**Table 1:** CV_50_ values for selected oligomer species and charge states

Oligomer species	Charge state	CV_50_^a^
Monomer	2 +	> 150
Dimer	4 +	> 140
	5 +	100
Trimer	5 +	> 140
	6 +	110
	7 +	70
Tetramer	8 +	80
Hexamer	10 +	80

^a^CV_50_ represents the cone voltage at which the signal intensity of a given species decreases to 50% of its maximum value

For instance, higher charge states decompose at lower CVs compared to lower charge states of the same oligomer, as observed for Tri^7+^ (70 V), Tri^6+^ (110 V), and Tri^5+^ (> 140 V). This trend is consistent with theoretical expectations, as ions with higher charge states are accelerated more rapidly, resulting in increased kinetic energy and dissociation during transmission.

Additionally, a comparison of oligomers with the same charge state highlights the influence of oligomer size on stability. For example, the CV_50_ values show that Tri^5+^ (> 140 V) decomposes at a higher CV than Di^5+^ (100 V), indicating that larger oligomers are less prone to dissociation while carrying an equivalent number of charges.

**Fig. 4:**
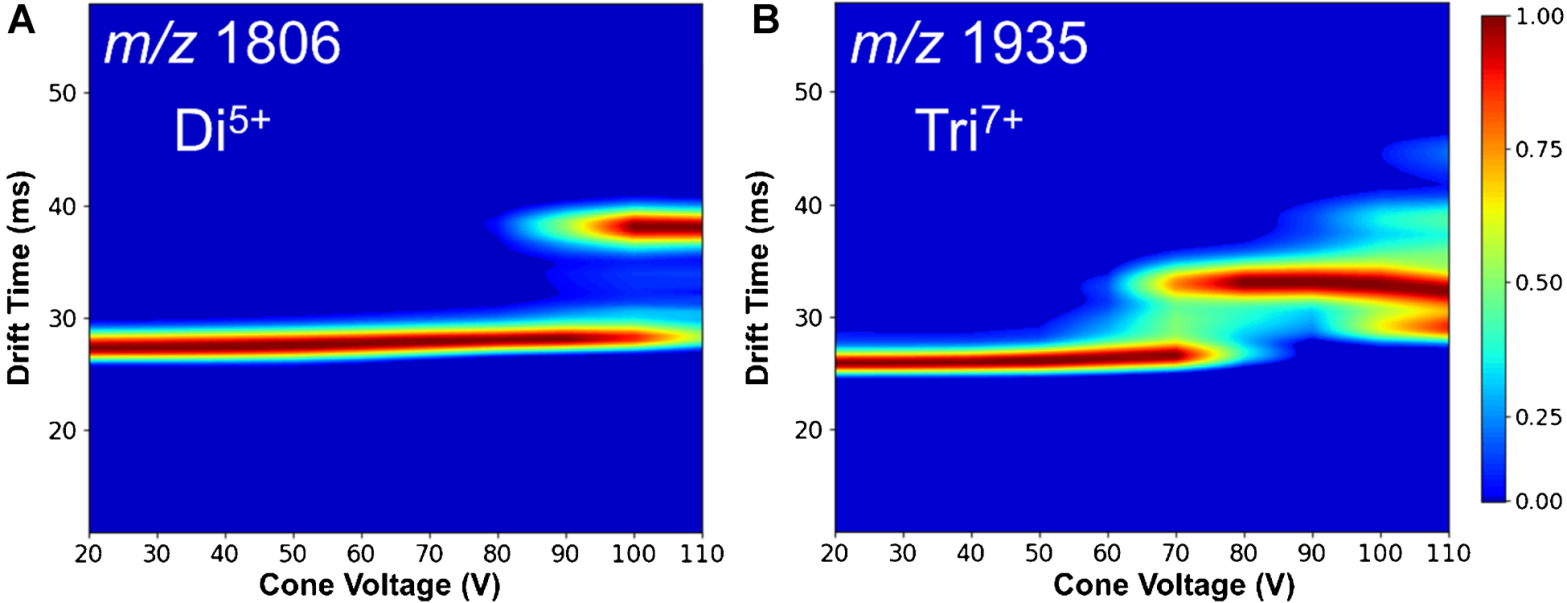
Arrival time distribution of Di^5+^ (**A**) and Tri^7+^ (**B**) as a function of cone voltage. At low cone voltages, the drift time remains stable, indicating a compact oligomeric structure. As the voltage increases beyond 80 V (Di^5+^) or 60 V (Tri^7+^), a shift in drift time is observed, suggesting the unfolding and the emergence of an extended conformation. The color scale indicates the relative intensity of the detected conformers

Besides causing the dissociation of non-covalent oligomers, in-source heating via increasing cone voltage can induce unfolding. This phenomenon, apparent in Fig. 2, is demonstrated by multiple conformations of Di^5+^ detected at 120 V CV. To explore this effect further, Fig. 4 shows the effect of cone voltage on the arrival time distribution of Di^5+^ and Tri^7+^ species. For Di^5+^, unfolding occurs at approximately 80 V while Tri^7+^ unfolds at 60 V. For both species, the shift in drift time reflects a transition from compact to more extended conformations. For Tri⁷⁺, an additional feature appears between the folded and unfolded conformations at 100 V, corresponding to a singly charged fragment generated by in-source fragmentation occurring at elevated cone voltages. The earlier onset of unfolding in Tri^7+^ suggests it is less stable than Di^5+^. These results highlight how increasing cone voltage affects the structural integrity of smaller Aβ(1–42) oligomers, further proving the significance of ion source parameter optimization.

Studying the effect of in-source activation on the analysis of Aβ(1–42) oligomers proved that CV is a critical parameter for maintaining the balance between ion desolvation and preserving non-covalent interactions during ion transmission. Using low CV values, such as 20 V, promotes retention of structural integrity and transmission of larger oligomers like Tet^8+^ and Tri^6+^, making them suitable for the analysis of intact higher-order species. However, the lower desolvation efficiency at low CV can limit sensitivity. Higher CV values, such as 120 V, significantly enhance the sensitivity of lower-order oligomers by promoting desolvation and adduct removal at the cost of larger oligomer dissociation. For detecting a broad range of oligomers while maintaining sensitivity and structural integrity, a moderate CV setting between 40 and 60 V is recommended. Such settings balance effective desolvation for higher sensitivity while minimizing the dissociation of higher-order oligomers.

### Source and trap pressure

The pressure within the instrument’s ion source is a crucial parameter for non-covalent complex ion desolvation and electrostatic focusing due to collisional cooling resulting from collisions of large-mass ions with neutral gas molecules [33, 39, 40, 49–51]. The cIMS instrument is equipped with a 0.9-mm sample cone aperture, which is larger than instruments from previous generations, such as Synapt G2 and Synapt HDMS, with sample cone diameters of 0.8 mm and 0.45 mm, respectively. Previous research revealed that due to higher pressure, larger sample cones promote collisional cooling and have a positive effect on large ion transmission [52].

The source region of the cIMS instrument consists of the ion block with a sample cone, the StepWave ion guide, and a segmented quadrupole ion guide, as shown in Fig. 1. Both the ion block and the initial segment of the StepWave operate at approximately 3.0 mbar, compared to about 1 mbar in older T-wave devices. This pressure is reported by the software as the backing pressure. The subsequent pressure, referred to as source pressure, corresponds to the pressure within the segmented ion guide and can be adjusted by modifying the ion guide gas flow in the operation software.

During the optimization procedure, we focused on the ion guide and the trap gas flows, which can both be readily changed within the operating software. The ion guide and trap gas flows were increased to 50 and 10 ml/min, respectively, to increase pressures within the source and trap regions. The change resulted in a seven-fold increase in pressure (1.3∙10^–2^ mbar) for the source and a two-fold increase for the trap region (5.1∙10^–2^ mbar) compared to default settings. As shown in Supplementary Figure S3, both adjustments increased signal intensity, with trap gas flow having a more significant effect. This optimization step created a milder pressure gradient, which is beneficial for transmitting non-covalent complex ions.

With the cone voltage optimized and a mild pressure gradient set to enhance ion cooling and transmission, our next focus was on fine-tuning the parameters of ion optics downstream of the ion source.

### Ion optics parameters

**Fig. 5:**
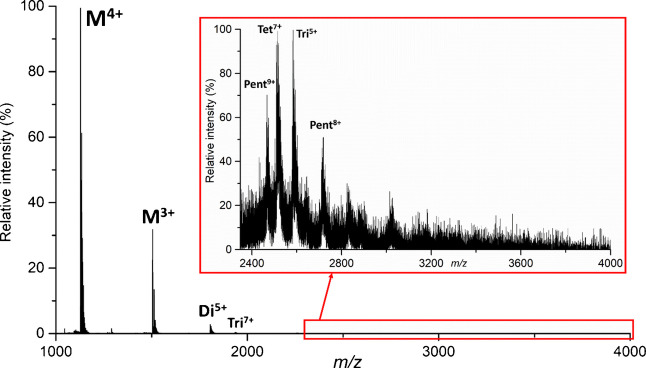
Mass spectra of Aβ(1–42) acquired at default instrument settings. Monomer, dimer, and trimer species were detected along with minor signals corresponding to tetramer and pentamer at higher *m*/*z* with an unresolved isotope pattern

By tuning the instrument settings, we aimed to improve the overall transmission efficiency of Aβ(1–42) oligomers. Spectra of Aβ(1–42) recorded prior to ion optics optimization (Fig. 5) reveal that the default instrument settings used for analysis of small molecules enabled the detection of monomer and dimer with minor signals of species up to pentamer with limited sensitivity.

The first and crucial part of the ion optics, propelling the ions from the ion source towards the quadrupole, is the StepWave ion guide (Fig. 1). This two-stage ion transfer device focuses ions while removing neutral species. The StepWave head has a potential gradient along the main instrument axis, while the body potential gradient is orthogonal.

Increasing the voltage gradient of StepWave, as well as the travelling wave height of the ion guide, helps with the effective extraction and transport of larger ions. Specifically, the StepWave body and head gradients were set to 20.0 V and 10.0 V, respectively. The ion guide parameters were as follows: TW velocity 300 m/s, TW height 10.0 V, and RF 700 V. Specifically, the increased ion guide RF voltage is essential for sensitive transmission of heavier ions on segmented quadrupole devices. The transfer cell RF voltage was also increased to 800V. Mass spectra showing the effect of these optimization steps are shown in Supplementary Figures S4 and S5.

The quadrupole settings were left at default values as detailed in Table S1, except the MS profile, which was set to *m/z* 1000, 2000, and 4000 to enable broader transmission of higher *m/z* ions and to suppress signals from lower *m/z* ions.

### Ion mobility parameters

Optimization of IMS parameters was a crucial step for the detection of a broader range of oligomers. The main components of the IMS region of the cIMS instrument are pre-array store, array, racetrack, and post-array store. The pre-array store retains the ions prior to injection. Pre-array store gradient affects the injection of the ions into the multifunctional array and further to the closed-loop electrode array (racetrack). Lower gradients are beneficial for transporting large complexes, while smaller *m/*z analytes require higher gradients. The array is a device with a set of microelectrodes that can generate travelling waves in three directions. Therefore, the array can direct the ions into the pre-array store, the IM cell, or the post-array store.

As mentioned previously, the single-pass cIMS method used in this study consisted of three steps. First, ions were injected into the multifunctional array from the pre-array store (10 ms). Next, the multifunctional array was set to propel the ions onto the racetrack for IM separation (5 ms). Finally, the array was set to eject the separated ions from the racetrack toward the transfer region, followed by the detector.

**Fig. 6:**
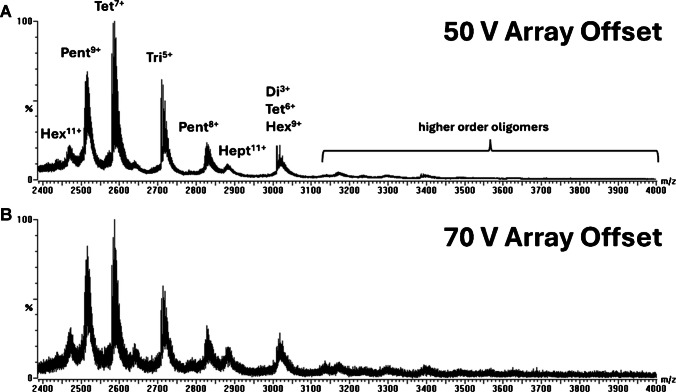
Mass spectra of Aβ(1–42) acquired at array offset and racetrack bias set to 50 V (**A**) and 70 V (**B**)

The parameters that heavily influence the detection of Aβ oligomers, array offset, and racetrack bias were lowered from 70 to 50 V for increased sensitivity (Fig. 6). Array offset and racetrack bias had to be set to equal values to avoid splitting peaks in IMS. A detailed description of this phenomenon was previously published [43].

**Fig. 7:**
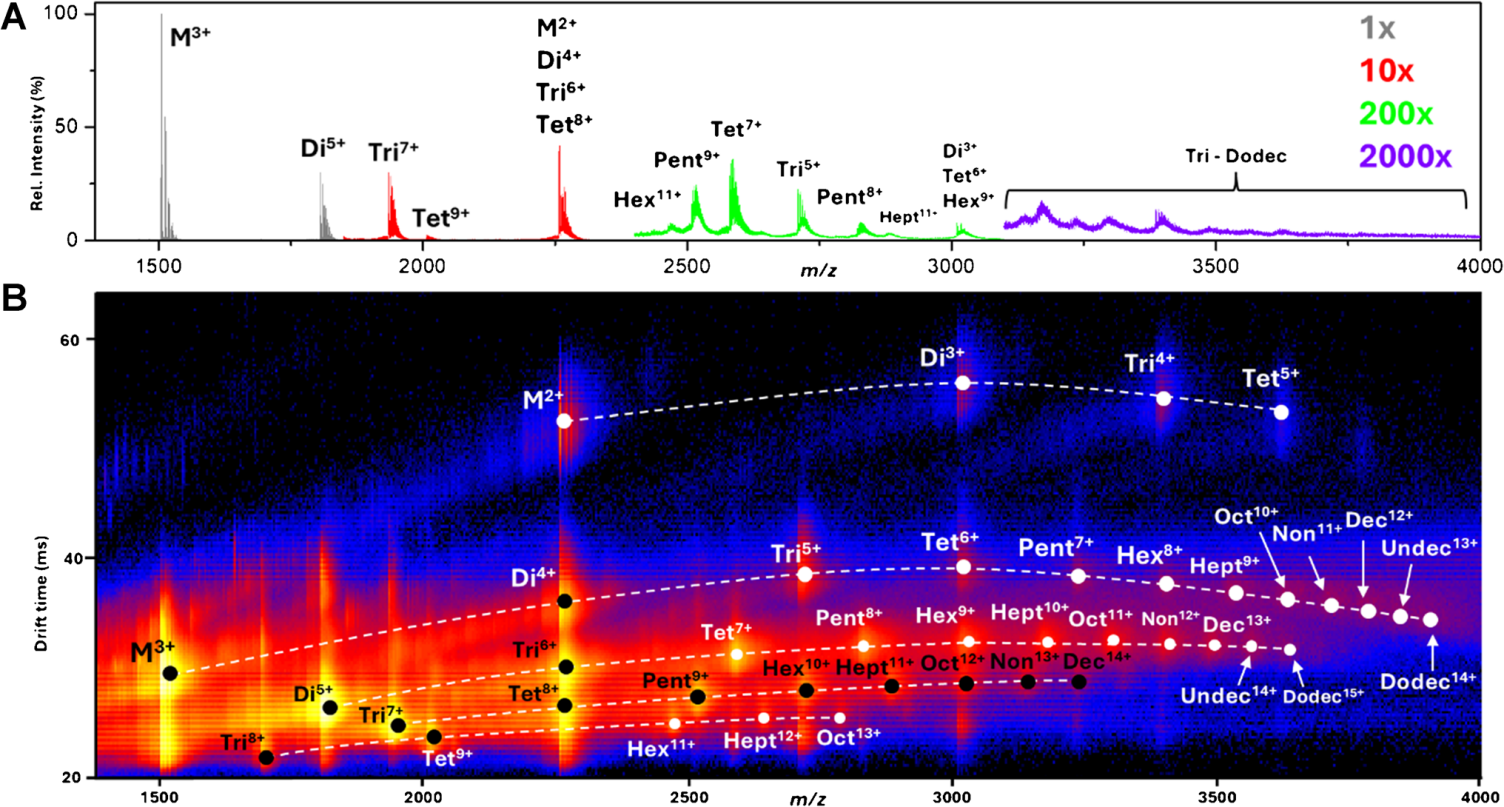
**A** Mass spectra and **B** mobilogram of Aβ(1–42) sample at 22 pmol/µl in water after 24-h incubation acquired with optimized cIMS method. The magnification of selected regions of the mass spectra is depicted by different colors. Signals of oligomers in multiple charge states ranging from dimer up to dodecamer were detected. The color coding of the heatmap describes the intensity on a logarithmic scale—black depicts no signal, blue noise, red low signal, and yellow high signal. Characteristic series of ions are connected by lines. This data shows successful detection and separation of Aβ(1–42) oligomers across multiple charge states, providing information about the oligomer distribution

The optimization of cone voltage and ion transmission parameters allowed us to preserve the native conformations of Aβ(1–42) oligomers, which were critical in detecting distinct species. Figure 7A shows the mass spectrum acquired at optimized conditions. Using the optimized settings and 20 V CV, oligomers ranging from dimer to dodecamer were detected by cIMS. Most of the oligomers were detected at multiple charge states. The two-dimensional mobilogram plot (Fig. 7B) shows the separation of oligomer ions into distinct series based on size and charge. The position of the ion in the series helps identify the oligomeric species in combination with the analysis of isotope patterns. The key parameters influencing Aβ(1–42) oligomer ion transmission efficiency are summarized in Table 2, while a comprehensive list of all instrumental settings is provided in Supplementary Tables S1 and S2.

**Table 2:** Critical parameters influencing Aβ(1–42) oligomer ion transmission

Instrument region	Parameter	Value
StepWave	Body gradient (V)	20
	Head gradient (V)	10
	Ion guide RF (V)	700
Cyclic IMS	Racetrack bias (V)	50
	Array offset (V)	50
Collision cells	Trap collision energy (V)	10
	Transfer collision energy (V)	10
	Collision gas 1 (ml/min)	10
	Transfer RF (V)	800

This method can serve as a basis for more complex experiments. For multi-pass separations, most of the parameters optimized for single-pass cIMS are directly applicable, except for separation time. More advanced experiments, such as drift-time-based ion selection, involve storing part of the ion mobility-separated species in the pre-array store and subsequently re-injecting them onto the racetrack. These experiments require additional optimization, particularly to ensure the efficiency of the re-injection step while minimizing ion activation or structural changes in the stored species. Fine-tuning parameters such as pre-array gradient and bias would be essential.

To further illustrate the versatility and applicability of the cIMS platform and our optimized method, we employed collision-induced unfolding to probe Aβ(1–42) oligomer stability and conformation.

### Collision-induced unfolding

Collision-induced unfolding (CIU) [53] was used in this study to characterize Aβ(1–42) oligomers using an optimized cIMS method. In a CIU experiment, ions isolated based on *m/z* are subjected to gradually increasing collisional energy in the trap region to induce conformational changes. Ion mobility measurement is then used to directly monitor structural changes. This approach provides deeper insights into the conformational dynamics and stability of detected Aβ(1–42) oligomers.

A key advantage of CIU compared to the in-source activation experiment illustrated in Fig. 4 is that in CIU, ions are first isolated and then activated in the trap collision cell. In contrast, in-source activation by CV takes place before the quadrupole, potentially generating interfering dissociation products prior to isolation.

**Fig. 8:**
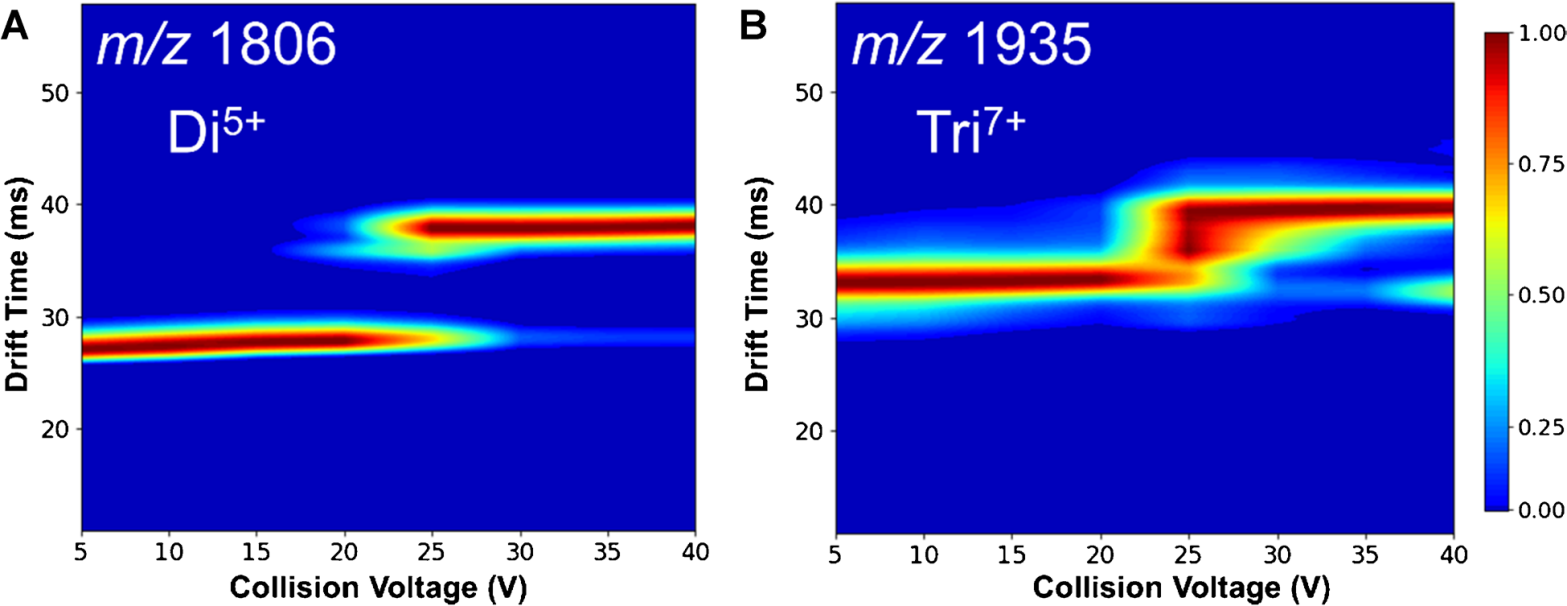
Collision-induced unfolding (CIU) of Aβ(1–42) oligomers detected by cIMS at 80 V CV. **A** CIU profile of the Di^5+^ (*m*/*z* 1806) species shows unfolding at around 20 V, indicating a transition from a compact to a more extended conformation. **B** The Tri^7+^ (m/z 1935) species show a similar trend but with unfolding starting at higher collision energy (CIU_50_ = 25 V), suggesting higher stability. The signal emerging at around 35 V with a median drift time of 32 ms is formed by a fragment ion with an overlapping *m*/*z* value

Our focus was on the collision-induced unfolding of Aβ(1–42) Di^5+^ and Tri^7+^ oligomer ions as they are most prevalent. Figure 8 displays the CIU plot for both species, showing conformation changes as a function of increasing collision voltage. At lower collision voltages, the dimer (Fig. 8A) shows a relatively narrow distribution pointing to a compact structure. The dimer ion starts to unfold at around 17 V collision voltage with the formation of a more extended conformer with a wider distribution. The CIU_50_ value can be used to describe the unfolding midpoint, defined as the voltage at which 50% of the initial conformer undergoes unfolding. In the case of the Di^5+^ species, the CIU_50_ is 22 V.

In comparison, the Tri^7+^ ion unfolds at higher collision voltages with CIU_50_ at 25 V. Moreover, the unfolding pattern of Tri^7+^ shows that unfolding occurs through transient unstable species, represented by the broad distribution of ATD at 25 V. The Di^5+^ unfolding by CID follows a similar trend as unfolding caused by in-source activation (Fig. 3), confirming the importance of proper optimization of the source parameters to avoid changes in oligomer composition and conformation.

These results align with the in-source activation by CV illustrated in Fig. 4. Both CIU and in-source activation show similar unfolding patterns, differing in their potential scales. In both cases, the initial Di^5+^ conformer, with an ATD of 27 ms, transitions to an extended conformer with an ATD of 38 ms. Unfolding induced by CV required a higher potential of approximately 90 V, compared to just 20 V for CIU performed in the trap collision cell.

In the case of Tri^7+^, the initial CIU conformer detected at 33 ms corresponds to the extended species formed at around 70 V CV. This effect can be attributed to the higher CV of 80 V used in this experiment to improve signal intensity. Moreover, the CIU of Tri^7+^ shows further unfolding of the species at 33 ms to even more extended conformation at 40 ms through a transient species. This conformation was not formed by in-source unfolding, probably due to milder activation. The higher number of detected Tri^7+^ conformations compared to Di^5+^ suggests higher conformational flexibility of Tri^7+^ likely resulting from its higher number of subunits.

Previous CIU analysis of Aβ(1–42) oligomers by Lieblein et al. [25], performed on an earlier-generation linear TWIMS instrument, reported a CIU₅₀ of 85 eV for the Di^5^⁺ species. In comparison, our cIMS measurements yield a CIU₅₀ of 138 eV. Such a difference can be partly attributed to differences in ion optics design between the platforms. Importantly, the cyclic instrument allows gentler transmission conditions, resulting in lower in-source activation and reduced ion heating prior to IM separation. This interpretation is supported by the observation that at 25 eV, Lieblein et al. detected both folded and partially unfolded conformations, whereas in our data, only the compact conformation is present at this energy, with the unfolded state emerging between 75 and 100 eV. Despite differences in absolute activation energies, the overall unfolding characteristics are highly similar between the studies, underscoring the robustness of the TWIM platform. Notably, the cIMS instrument provides slightly higher resolution in single-pass mode, with the option to employ multiple passes to further enhance separation.

### Gas phase stability in multi-pass mode

**Fig. 9:**
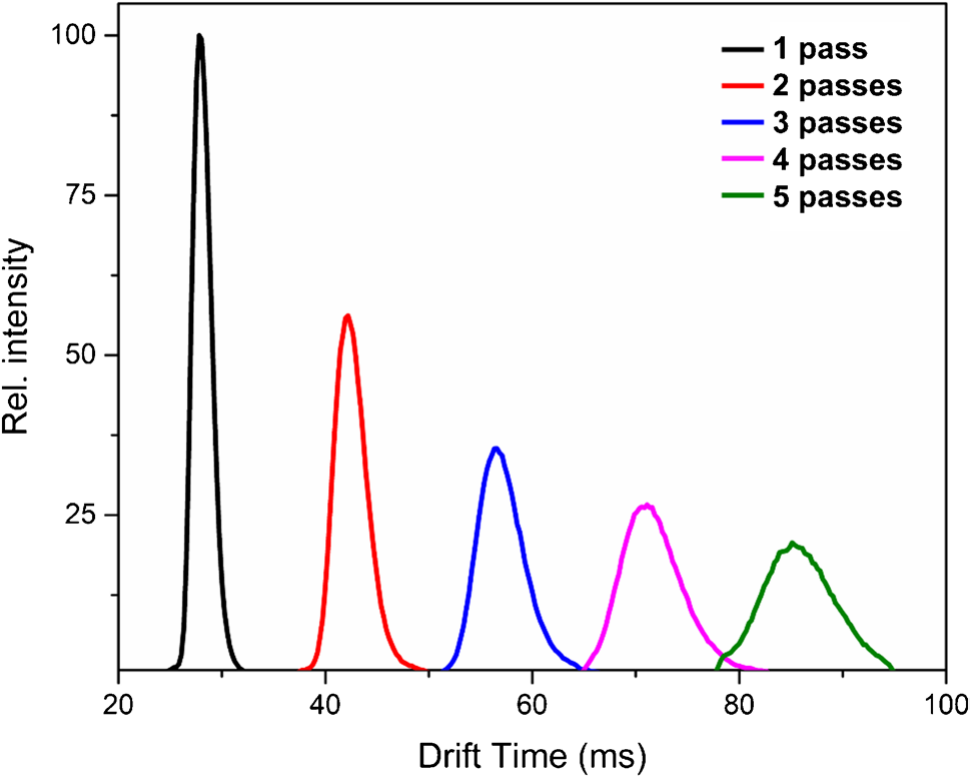
Arrival time distributions of the *m/z* 1806 showing gas-phase stability of Di^5+^ oligomer species during multi-pass separation. Quadrupole-isolated ions were subjected to one to five passes through the cyclic ion mobility cell

The effect of multiple passes in the cyclic ion mobility cell on the transmission of labile oligomer ions was studied by subjecting the Di^5+^ species to an increasing number of separation cycles. Ions were first isolated in the quadrupole, injected into the ion mobility cell, and separated over progressively longer separation times. As shown in Fig. 9, extended residence of oligomer ions within the mobility region led to a mild gradual decrease in signal intensity and broadening of the ATD peak. The transmission loss, quantified from peak areas, was ~6.2% per pass and followed a pattern similar to that previously reported for native-like cytochrome c + 7 ions [42], which are highly susceptible to structural rearrangements [54]. The observed peak broadening reflects partial activation of the ions, resulting in minor conformational relaxation, combined with increased diffusion during extended separation; however, unlike Di^5+^ in CIU experiments, no structural rearrangement is observed, allowing for multi-pass experiments for structural investigations.

## Conclusion

This work provides a detailed description of the parameters affecting the detection of Aβ(1–42) oligomers using cyclic ion mobility-mass spectrometry, addressing a critical need for accurate characterization of these key drivers of Alzheimer’s disease progression and pathology. We focused on optimizing key ion source settings and ion optics to enhance the sensitivity and reliability of detecting a broad spectrum of oligomeric species while preserving their structural integrity. Furthermore, we investigated the effect of in-source activation on the structural integrity of the studied non-covalent complex species. Specifically, lower cone voltage values (e.g., 20 V) are beneficial for transmission and detection of a wider range of oligomers due to lower activation, while limiting the desolvation and declustering, leading to lower signal intensities of monomers and small oligomers. Conversely, higher CV values (e.g., 120 V) enhanced the signal intensity for smaller species but led to unfolding and dissociation of larger oligomers. A careful balance between desolvation and excessive activation is required for reliable analysis of a broad spectrum of oligomeric species while maintaining their native conformations and sufficient signal intensity. Further adjustments, including lowered array offset and racetrack bias, enabled the efficient transmission of higher *m/z* species, improving the detection of larger oligomers. These optimizations yielded significant improvement in sensitivity and gains in signal intensity, with detection enhancements of threefold for monomers, 72-fold for dimers, and 64-fold for trimers.

Comparison of CIU patterns with earlier TWIMS platforms shows that, despite differences in absolute CIU_50_ values due to instrument and method-specific activation, the unfolding pathways remain consistent. This highlights the robustness of the TWIM platforms.

Multi-pass experiments revealed moderate ion transmission loss (~ 6.2% per pass) for dimer ions and ATD broadening due to diffusion and ion activation over extended flight paths. These observations outline the practical limits of multi-pass separation for Aβ oligomers while demonstrating its feasibility when higher separation efficiency is needed. The successful detection of oligomers, ranging from dimers to dodecamers, demonstrates the versatility of the cIMS platform, offering strong ion mobility capabilities and the ability to analyze such complex biomolecular systems and matrices. Our findings provide a background for analyzing non-covalent Aβ(1–42) oligomers by cyclic ion mobility-mass spectrometry. We believe that the information presented here can serve as a technical foundation for future research focused on understanding the pathology of Alzheimer’s disease and the assessment of potential therapeutics, as well as other biological systems.

## Supplementary Information

Below is the link to the electronic supplementary material.

ESM 1 (1.79 MB DOCX)

## Data Availability

The data are available from the corresponding author on request.

## Abbreviations

Aβ: Amyloid-beta
AD: Alzheimer’s disease
cIMS: Cyclic ion mobility-mass spectrometry
CV: Cone voltage
ECD: Electron-capture dissociation
HFIP: 1,1,1,3,3,3-Hexafluoro-2-propanol
LC-MS: Liquid chromatography-mass spectrometry
ATD: Arrival time distribution
TWIM: Travelling wave ion mobility
RF: Radio frequency
TOF: Time-of-flight
CIU: Collision-induced unfolding
