# Supplementary material for: Analysis of amyloid beta oligomers by cyclic ion mobility-mass spectrometry
Source: Anal Bioanal Chem. 2026 Jan 31;418(8):2273–86. doi: 10.1007/s00216-026-06349-w (PMC13065575; doi:10.1007/s00216-026-06349-w)
Supplement: Supplementary file 1 — (1.79 MB DOCX) [file 216_2026_6349_MOESM1_ESM.docx]

**Supporting Information for**

**Analysis of Amyloid Beta Oligomers by Cyclic Ion Mobility-Mass Spectrometry**

Mikuláš Vlk^1,2^, Alexander Muck^3^, John A. Hey^4^, Jean F. Schaefer^4^, Martin Hubálek^2^,
Josef Cvačka*****^1,2^

^1^Department of Analytical Chemistry, Faculty of Science, Charles University, Hlavova 2030/8, 128 00, Prague, Czech Republic

^2^ Institute of Organic Chemistry and Biochemistry of the Czech Academy of Sciences, Flemingovo náměstí 542/2, 16600, Prague, Czech Republic

^3^ Waters Corporation, Stamford Avenue, Altrincham Road, Wilmslow SK9 4AX, United Kingdom

^4^ Alzheon, Inc., 111 Speen Street, Framingham, MA 01701, USA

* Correspondence**:** josef.cvacka@uochb.cas.cz

The Supporting Information includes following items:

Table S1 – Optimized MS method parameters.

Table S2 – Cyclic ion mobility method parameters and sequence.

Figure S1 – Mass spectra of samples Aß(1-42) sample treated with HFIP + 3% NH3 at 22 µmol/l analyzed by cIMS (A) prior to incubation (t=0 h) and (B) after 24 h incubation at 21°C.

Figure S2 – Mass spectra of Aß(1–42) samples at 22 µmol/L, recorded at (A) 20 V CV and (B) 120 V CV.

Figure S3 Higher mass range mass spectra of Aß(1-42) sample recorded at multiple gas flow settings.

Figure S4 – Higher mass range mass spectra of Aß(1-42) sample recorded at (A) 10 V Trap and Transfer collision energy. (B) 6 V Trap and 4 V Transfer collision energy.

Figure S5 - Higher mass range mass spectra of Aß(1-42) sample recorded at (A) 700 V Ion guide RF and 800 V Transfer RF. (B) 250 V Ion guide RF and 300 V Transfer RF.

*Table S1 – Optimized MS method parameters.*

| **StepWave** |  | **Quad/MS Profile/DRE** |  | **Trap** |  | **Cyclic IMS** |  | **Transfer** |  | **RF** |  |
| --- | --- | --- | --- | --- | --- | --- | --- | --- | --- | --- | --- |
| Body Gradient (V) | 20 | Ion Energy (V) | 1,0 | Trap TW Velocity (m/s) | 300 | Helium Entrance (V) | 10,0 | Pre ECD Gradient (V) | 2,0 | StepWave RF (V) | 200 |
| Head Gradient (V) | 10 | Pre-filter (V) | 5,0 | Trap TW Pulse Height (V) | 0,1 | Helium Cell Bias (V) | 30,0 | Pre ECD Bias (V) | 4,0 | Ion Guide RF (V) | 700 |
| Ion Guide 1 Offset (V) | 5,0 | Trap Collision Energy (V) | 10 | Trap Entrance (V) | 1,0 | Helium Exit DC (-V) | 10,0 | Pre Trans Gradient (V) | 1,0 | Trap RF (V) | 500 |
| Ion Guide 2 Offset (V) | 0,5 | Transfer Collision Energy (V) | 10 | Trap Bias (V) | 1,0 | Pre IMS Reference (V) | 0,0 | Pre Trans Bias (V) | 0,0 | Driftcell RF (V) | 300 |
| Diff Ap 2 (-V) | 0,1 | DRE Attenuate | Off | Trap DC (V) | -1,0 | Racetrack Bias (V) | 50,0 | Transfer Entrance (V) | 1,0 | Pre/Post Array RF (V) | 350 |
| IG TW Velocity (m/s) | 300 |  |  | Trap Exit (V) | 1,0 | Repeller (V) | 100,0 | Transfer Gradient (V) | 1,0 | Cyclic RF (V) | 250 |
| IG TW Pulse Height (V) | 10,0 |  |  | Post Trap Gradient (V) | 7 |  |  | Transfer Exit (V) | 10,0 | Transfer RF (V) | 800 |
| Ion Guide Gas (ml/min) | 50 |  |  | Post Trap Bias (V) | 35 |  |  |  |  | Transfer RF Gain (V) | 5 |
|  |  |  |  | Collision Gas Setting | Gas 1 to Trap/Transfer |  |  |  |  | Ion Guide RF Ramp | OFF |
|  |  |  |  | Collision Gas 1 (ml/min) | 10 |  |  |  |  | MS/MS Ramp Type | Single |
|  |  |  |  | Collision Gas 2 (ml/min) | 0 |  |  |  |  | MS/MS Ramp Mode | Automatic |

*Table S2 – Cyclic ion mobility method parameters and sequence.*

| **Travelling wave parameters** | | **Sequence** | | | |
| --- | --- | --- | --- | --- | --- |
|  |  | **Parameter** | **Inject** | **Separate** | **Eject and Acquire** |
| Cyclic TW Velocity (m/s) | 375 | Time (ms) | 10 | 5 | automatic |
| Array TW Velocity (m/s) | 500 | Time Abs (ms) | 10 | 15 | automatic |
| TW static height (V) | 25,0 | Pre Array Gradient (V) | 75 | 75 | 75 |
|  |  | Pre Array Bias (V) | 75 | 75 | 75 |
|  |  | Array Entrance (V) | 10 | 30 | 50 |
|  |  | Wave Height (V) | 4 | 25 | 15 |
|  |  | Array Offset (V) | 40 | 50 | 45 |
|  |  | Array Mode (V) | Forward | Sideways | Forward Eject |
|  |  | Array Exit (V) | 50 | 30 | 5 |
|  |  | Post Array Gradient (V) | 35 | 35 | 35 |
|  |  | Post Array Bias (V) | 10 | 10 | 10 |


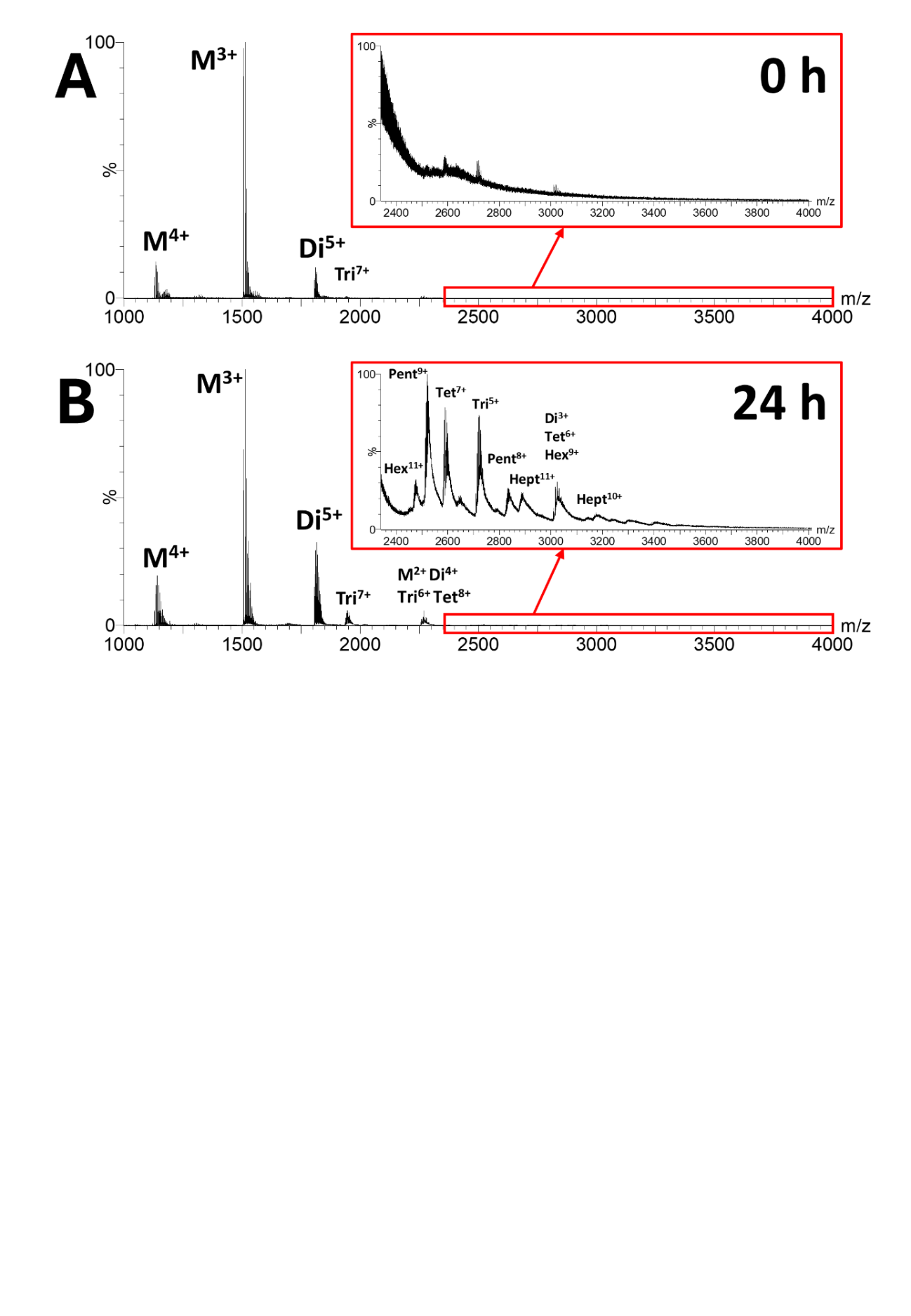


*Figure S1 –* *Mass spectra of samples Aß(1-42) sample treated with HFIP + 3% NH_3_ at 22 µmol/l analyzed by cIMS* ***(A)*** *prior to incubation (t=0 h) and* ***(B)*** *after 24 h incubation at 21°C.*

*
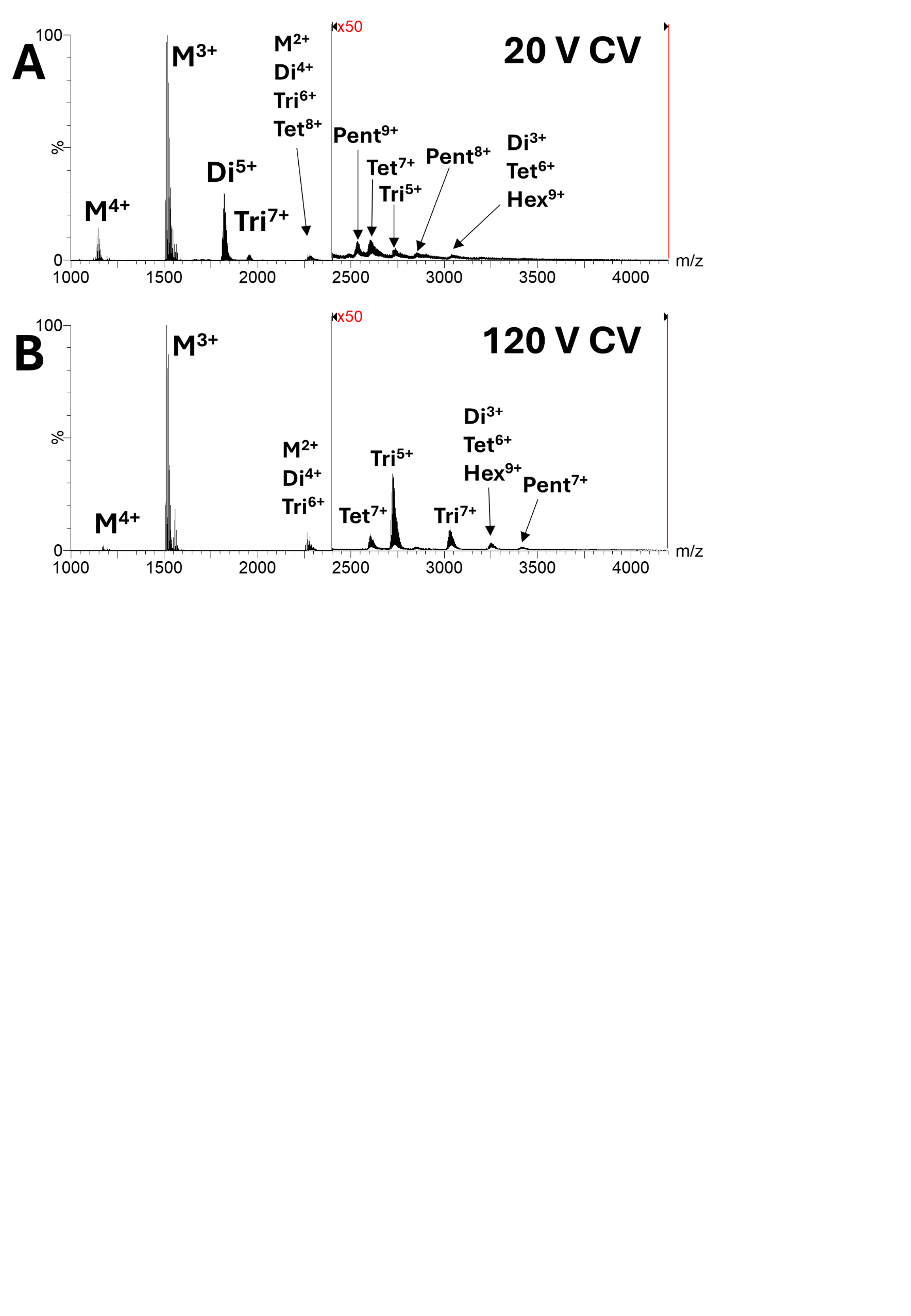
*

*Figure S2 – Mass spectra of Aß(1–42) samples at 22 µmol/L, recorded at* ***(A)*** *20 V CV and* ***(B)*** *120 V CV. The red-marked regions of the spectra indicate a 50× magnification.*


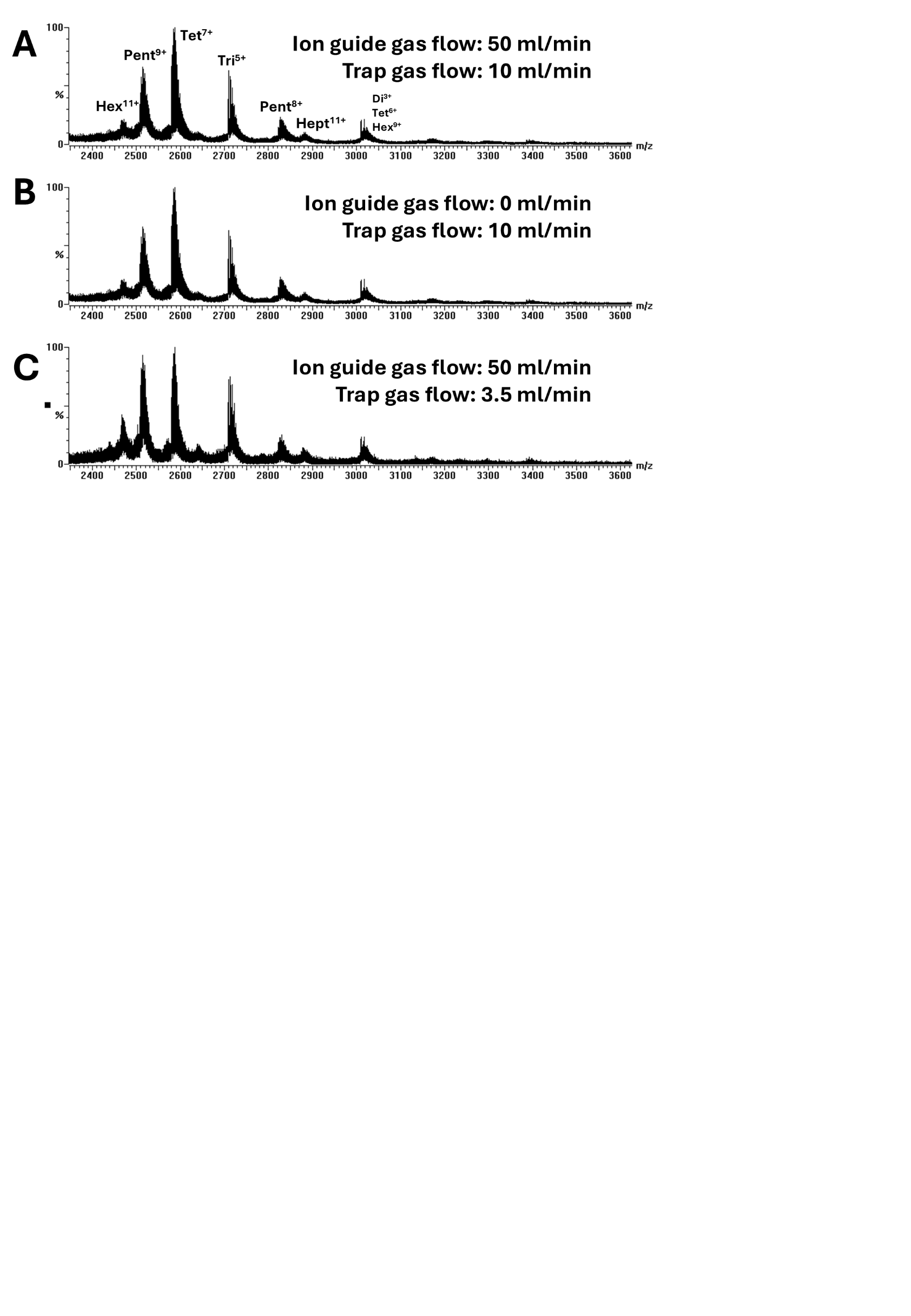


*Figure S3 Higher mass range mass spectra of Aß(1-42) sample recorded at multiple gas flow settings.*


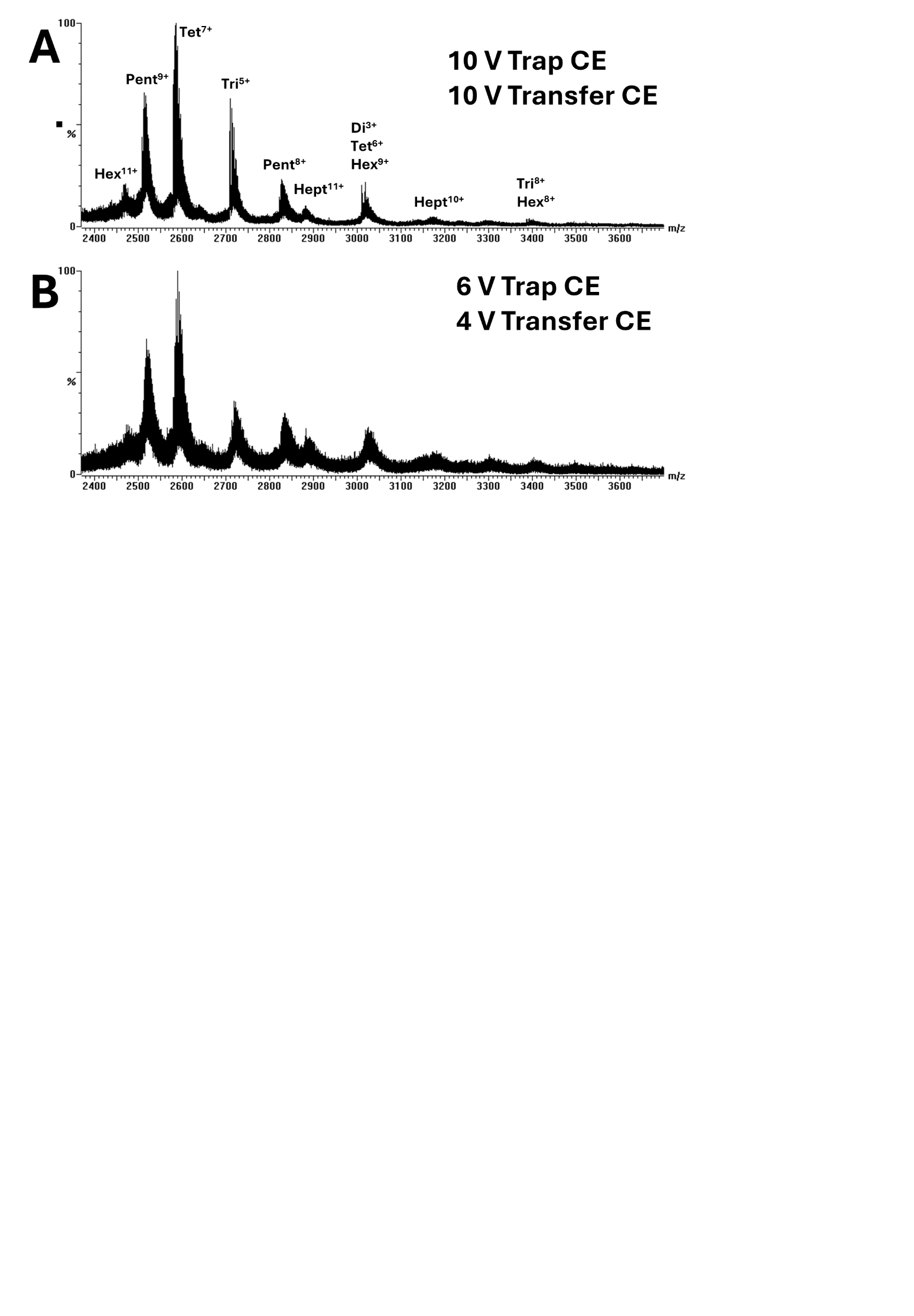


*Figure S4 – Higher mass range mass spectra of Aß(1-42) sample recorded at* ***(A)*** *10 V Trap and Transfer collision energy.* ***(B)*** *6 V Trap and 4 V Transfer collision energy.*


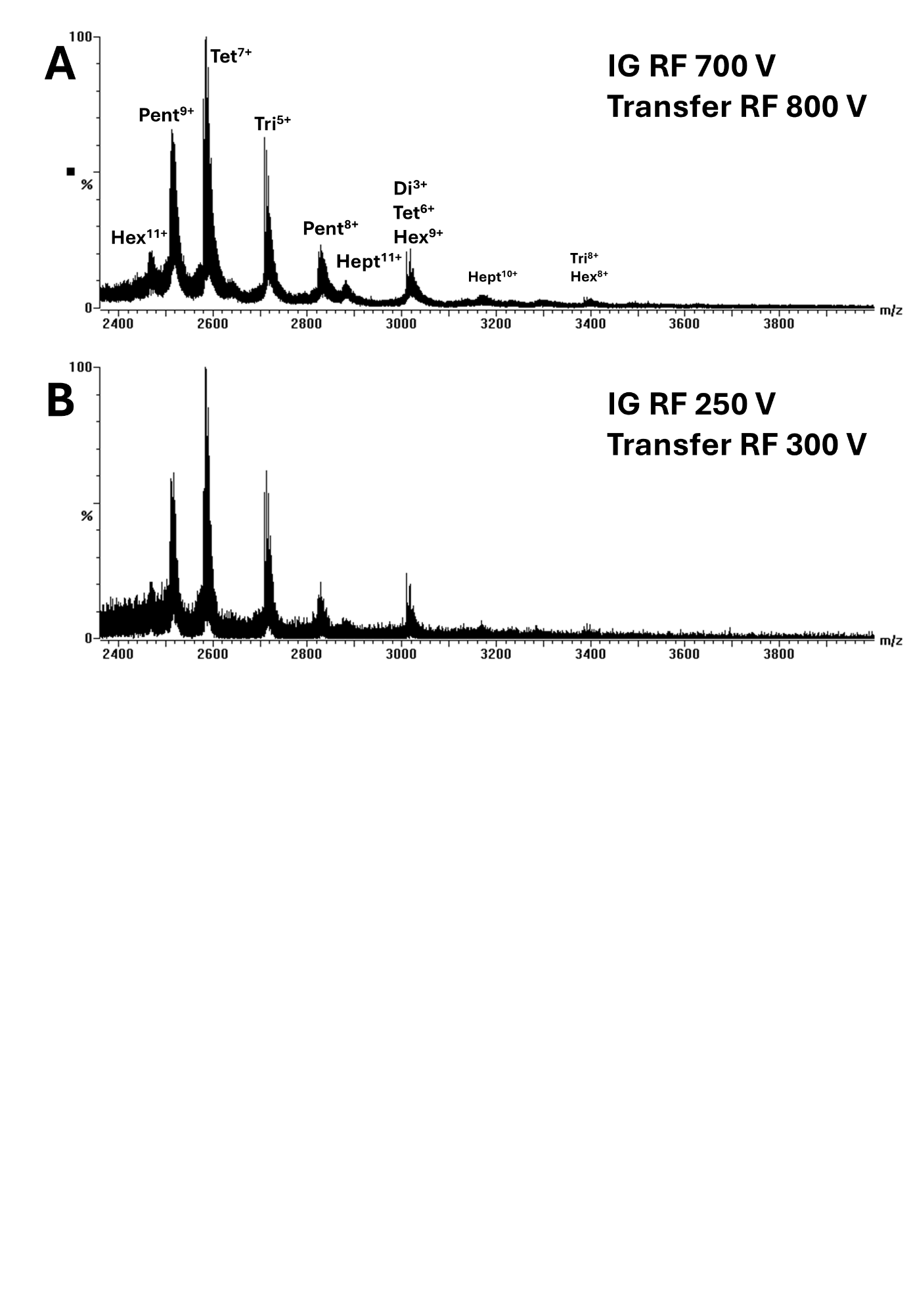


*Figure S5 - Higher mass range mass spectra of Aß(1-42) sample recorded at* ***(A)*** *700 V Ion guide RF and 800 V Transfer RF.* ***(B)*** *250 V Ion guide RF and 300 V Transfer RF.*
